# Time trends and risk factors for perioperative complications in total ankle arthroplasty: retrospective analysis using a national database in Japan

**DOI:** 10.1186/s12891-016-1299-x

**Published:** 2016-10-28

**Authors:** Takumi Matsumoto, Hideo Yasunaga, Hiroki Matsui, Kiyohide Fushimi, Naohiro Izawa, Tetsuro Yasui, Yuho Kadono, Sakae Tanaka

**Affiliations:** 1 Department of Orthopaedic Surgery, Faculty of Medicine, The University of Tokyo, 7-3-1 Hongo, Bunkyo-ku, Tokyo, 113-8655 Japan; 2 Department of Clinical Epidemiology and Health Economics, School of Public Health, The University of Tokyo, 7-3-1 Hongo, Bunkyo-ku, Tokyo, 113-8655 Japan; 3 Department of Health Policy and Informatics, Tokyo Medical and Dental University Graduate School, 1-5-45 Yushima, Bunkyo-ku, Tokyo, 113-0034 Japan; 4 Department of Orthopaedic Surgery, Teikyo University Mizonokuchi Hospital, 3-8-3 Mizonokuchi, Takatsu-ku, Kawasaki, 213-8507 Japan

**Keywords:** Ankle arthrodesis, Ankle arthroplasty, Ankle fusion, Low-volume hospitals, Outcomes, Trends

## Abstract

**Background:**

Total ankle arthroplasty (TAA) has become increasingly popular worldwide as an alternative to ankle arthrodesis for surgical treatment of end-stage ankle arthritis. The aim of this epidemiological study, using a national inpatient database in Japan, was to describe the volume, utilization, patient characteristics, and temporal trends regarding these procedures in Japan, and to identify the risk factors associated with perioperative adverse events in TAA.

**Methods:**

This was a population-based, retrospective cohort study. We retrospectively identified 2775 patients in the Diagnosis Procedure Combination database who underwent ankle arthrodesis or TAA for ankle arthritis at 559 hospitals in Japan from 2007 to 2013. Information on sex, age, main diagnosis, use of blood transfusion, duration of anesthesia, length of hospital stay, in-hospital mortality, hospitalization costs, additional procedures after primary surgery, and use of negative pressure wound therapy was extracted. Multivariable logistic regression analysis was performed to analyze the effect of various factors on the incidence of perioperative adverse events in TAA, including additional procedure during hospitalization, negative pressure wound therapy, blood transfusion, and in-hospital death.

**Results:**

We identified 465 patients who underwent TAA and 2310 patients who underwent ankle arthrodesis. There was no apparent increase in the proportion of TAAs performed during the survey period. Patients undergoing TAA tended to be older, female, and have rheumatoid arthritis compared with those undergoing ankle arthrodesis. Patients undergoing TAA had shorter length of stay, higher hospitalization costs, and more blood transfusions compared with those undergoing ankle arthrodesis. Lower hospital volume and shorter anesthesia time were associated with higher rates of adverse events after TAA.

**Conclusions:**

Despite an increase in the popularity of TAA internationally, the number of TAAs performed remains low in Japan. Lower hospital volume and anesthesia time were associated with higher rates of perioperative adverse events after TAA.

**Level of evidence:**

IV, Cross-sectional study

## Background

Forty years have passed since the introduction of the first-generation total ankle arthroplasty (TAA), which ended in failure because of high rates of aseptic loosening and pain. Refinement of implant design by adoption of mobile bearings and technological advances has gradually improved the clinical outcomes after TAA. A recent systematic review of recent TAA studies, reported an overall survivorship of 89 % at 10 years [1]. Based on improved clinical outcomes, TAA has become increasingly popular as an alternative to arthrodesis, which has long been the gold standard for treating end-stage ankle arthritis. A previous report using the United States Nationwide Inpatient Sample data contained data from five to eight million hospital stays and discharges from > 1,000 hospitals sampled to approximate a 20 % stratified sample of United States community hospitals. This sample showed an approximately 6-fold increase in TAA utilization in the previous decade, from 0.13 per 100,000 population in 1998 to 0.84 per 100,000 population in 2010 [2]. Another study from the United States using the Medicare database included 5871 patients who underwent TAA and 29532 patients who underwent ankle arthrodesis between 1991 and 2010; this study reported an increase in TAA volume from 72 procedures in 1991 to 888 in 2010 [3]. The study also revealed that the percentage of all United States hospitals performing TAA increased by approximately 4-fold from 3.1 % in 1991 to 12.6 % in 2010, while the proportion performing ankle arthrodesis remained unchanged. Although the sample sizes were smaller than those in the United States studies, joint registry data from northern European nations also showed a more than 2-fold increase in TAA in a recent decade [4, 5]. Joint registry data from New Zealand demonstrated an approximately 4-fold increase in TAA from 26 arthroplasties in 2003 to 113 arthroplasties in 2013 [6].

Because there is a steep learning curve for the TAA procedure [7–11], improved outcomes and decreased complications would be expected by virtue of the global growth in the number of procedures performed [12]. Although there has been an increase in the number of TAA procedures performed around the world, TAA is still considered a fairly rare procedure in Japan. There have been no published studies revealing epidemiological data on TAA utilization within Japan.

Although TAA has been recently popularized worldwide, it is still accompanied by higher rates of complications compared with total hip and knee arthroplasties [1, 13–15]. Surgeon understanding of the complications and their risk factors is important to provide good clinical outcomes; however, there is limited evidence available in the literature about risk factors for perioperative complications after TAA.

The purpose of this study was (i) to assess recent trends in the use of TAA compared with ankle arthrodesis in Japan, and (ii) to identify the risk factors associated with perioperative complications in TAA using the Diagnosis Procedure Combination (DPC) database, a large national inpatient database in Japan.

## Methods

### Data source

The DPC database is a national administrative claims and discharge database on acute-care inpatients in Japan. All 82 academic hospitals in Japan are obliged to adopt this system, while community hospitals participate on a voluntary basis. The numbers of participating hospitals were 898, 855, 901, 980, 1,075, 1,057, and 1,061, in fiscal years 2007, 2008, 2009, 2010, 2011, 2012, and 2013, respectively. Data were collected during 6 months (from July 1 to December 31) between fiscal years 2007 and 2009, 9 months (from July 1 to March 31) in fiscal year 2010, and throughout the year (from April 1 to March 31) from fiscal year 2011 on. The numbers of admissions in the database were 2.65, 2.81, 2.78, 4.95, 7.14, 6.85, and 7.11 million in fiscal years 2007, 2008, 2009, 2010, 2011, 2012, and 2013, respectively. The number in 2013 represented approximately 50 % of all inpatient admissions to acute-care hospitals in Japan.

The DPC database includes the following: age and sex; diagnoses, comorbidities at admission, and complications after admission recorded with text data in the Japanese language and the International Classification of Diseases, Tenth Revision (ICD-10) codes; procedures coded using the Japanese original codes; duration of anesthesia; drugs and implants used; blood transfusion; length of hospital stay; in-hospital mortality; and hospitalization costs.

### Data extraction

Data were retrospectively collected on patients who received either primary TAA or ankle arthrodesis between 2007 and 2013. Patients who had a fracture from a traffic accident, pyogenic arthritis, Charcot disease, diabetic arthritis, or pigmented villonodular synovitis were excluded to focus on patients with ankle arthritis due to inflammatory pathology and osteoarthritis. Eligible patients were divided into osteoarthritis (OA) and rheumatoid arthritis (RA) groups. The outcomes included length of hospital stay (days), hospitalization costs, operative costs, use of blood transfusion, in-hospital mortality, additional procedures during hospitalization after primary surgery, and use of negative pressure wound therapy. Details of additional procedures were further divided into the following categories: wound treatment, skin graft of flap, and limb amputation. Perioperative adverse events were defined as the composite outcome, including additional procedure during hospitalization, negative pressure wound therapy, blood transfusion, and in-hospital death.

Based on the protocol by Quan et al. [16], each ICD-10 code of a comorbidity was converted to a score, and the sum of the scores, excluding the score for RA (=1), was used to calculate the patient’s Charlson Comorbidity Index [17]. Hospital volume was defined as the mean number of TAAs performed per year at each institution. Patients were dichotomized into low-volume (≤ 4 cases/year) and high-volume (≥ 5 cases/year) groups according to the only previous study reporting the influence of hospital volume on complications after TAA [18]. For analysis of hospitalization costs, 1 US dollar was assumed to be 100 Japanese yen.

### Statistical analysis

We performed univariate comparisons of explanatory variables using the chi-square test or analysis of variance, as appropriate. Multivariable linear regression or multivariable logistic regression analyses were performed for the comparison of outcomes between TAA and ankle arthrodesis to adjust for the differences in demographics. Multivariable logistic regression analysis was performed to compare the independent factors associated with perioperative adverse events after TAA, with adjustment for other variables. All demographic variables with a *P*-value less than 0.1 in the univariate analyses were entered into the multivariable logistic regression model. A *P*-value less than 0.05 was considered statistically significant, and Bonferroni adjustments were used to reduce the potential for false positives due to multiple comparisons. All statistical analyses were performed using SPSS version 19.0 (IBM Corp., Armonk, NY, USA).

## Results

We identified 465 patients who underwent TAA and 2,310 patients who underwent ankle arthrodesis at 559 hospitals during the survey period. The absolute numbers and proportions of these two procedures in each year are shown in Table 1. The proportion of TAAs fluctuated between 12 and 20 %.

**Table 1:** Numbers of patients and hospitals undergoing ankle arthrodesis or total ankle arthroplasty

Year	2007	2008	2009	2010	2011	2012	2013	Total
Ankle arthrodesis	152 (80.4 %)	223 (87.5 %)	183 (85.1 %)	293 (88.3 %)	440 (82.4 %)	524 (82.1 %)	495 (80.9 %)	2310 (83.2 %)
Total ankle arthroplasty	37 (19.6 %)	32 (12.5 %)	32 (14.9 %)	39 (11.7 %)	94 (17.6 %)	114 (17.9 %)	117 (19.1 %)	465 (16.8 %)
Total	189	255	215	332	534	638	612	2775
Data collection periods (months)	6	6	6	9	12	12	12	—
Number of enrolled hospitals in DPC	898	855	901	980	1075	1057	1061	—
Number of admissions in DPC (million)	2.65	2.81	2.78	4.95	7.14	6.85	7.11	34.29

Table 2 shows the patient backgrounds. The mean age and the proportion of female patients were significantly higher in the group who underwent TAA (69.1 years; 83.7 %) compared with those who underwent ankle arthrodesis (64.5 years; 70.7 %). The average age was significantly higher in the TAA group than the ankle arthrodesis group among OA patients, but was not significantly different among RA patients. Patients with TAA were more likely to have RA (38.5 vs 24.0 %). There was no significant difference in anesthesia time between the two groups.

**Table 2:** Comparison of patient characteristics and anesthesia time between ankle arthrodesis and total ankle arthroplasty groups

	Total (*n* = 2775)	Ankle arthrodesis (*n* = 2310)	Total ankle arthroplasty (*n* = 465)	*P*-value
Age (years), mean ± SD	65.7 ± 11.2	64.5 ± 11.4	69.1 ± 9.2	**< 0.001**
in osteoarthritis patients	66.3 ± 11.6	65.4 ± 11.8	72.1 ± 8.3	**< 0.001**
in rheumatoid arthritis patients	63.9 ± 9.9	63.8 ± 10.2	64.4 ± 8.6	0.452
Sex (female)	2022 (72.9 %)	1633 (70.7 %)	389 (83.7 %)	**< 0.001**
Body mass index (kg/m^2^), mean ± SD	24.4 ± 3.9	24.5 ± 4.0	24.2 ± 3.7	0.328
Smoking				
Nonsmoker	1931 (69.6 %)	1590 (68.8 %)	341 (73.3 %)	0.043
Ex or current smoker	323 (11.6 %)	284 (12.3 %)	39 (8.4 %)	
Missing	521 (18.8 %)	436 (18.9 %)	85 (18.3 %)	
Underlying diagnosis				
Osteoarthritis	2042 (73.6 %)	1756 (76.0 %)	286 (61.5 %)	**< 0.001**
Rheumatoid arthritis	733 (26.4 %)	554 (24.0 %)	179 (38.5 %)	
Charlson Comorbidity Index				
0	2472 (89.0 %)	2049 (88.7 %)	423 (91.0 %)	0.153
≥ 1	303 (11.0 %)	261 (11.3 %)	42 (9.0 %)	
Anesthesia time (minutes), mean ± SD	199 ± 91	199 ± 93	200 ± 84	0.127

The values in bold indicate significant differences at the 0.007 (=0.05/7) significance level

The TAA group had a significantly shorter average hospital stay compared with the ankle arthrodesis group, and total hospitalization costs and operative costs were significantly higher in the TAA group compared with the ankle arthrodesis group (Table 3); the differences in these variables were also significant after adjusting the differences in demographics (Table 3A, B). There were no significant differences in the performance of an additional procedure during hospitalization or in the use of negative pressure wound therapy between the ankle arthrodesis and TAA groups, and the differences were also not significant after adjusting for the differences in demographics (Table 3A, B). The proportion of patients receiving a blood transfusion was significantly higher in the TAA group than in the ankle arthrodesis group, although this difference was not significant after adjusting for the differences in demographics (Table 3A, B).

**Table 3:** Comparison of perioperative outcomes between ankle arthrodesis and total ankle arthroplasty groups (A), and adjusted coefficient of determination and odds ratio of major outcomes in total ankle arthroplasty group using ankle arthrodesis group as a control (B)

A.				
	Total (*n* = 2775)	Ankle arthrodesis (*n* = 2310)	Total ankle arthroplasty (*n* = 465)	*P*-value
Length of stay (days), mean ± SD	41.8 ± 28.2	42.8 ± 29.1	36.9 ± 23.6	**< 0.001** ^**a**^
Hospitalization costs (dollars), mean ± SD	16111 ± 8002	15123 ± 8085	21019 ± 7595	**< 0.001** ^**a**^
Costs of operation (dollars), mean ± SD	6196 ± 2635	5063 ± 2576	11827 ± 2927	**< 0.001** ^**a**^
Additional procedure during hospitalization	90 (3.2 %)	76 (3.3 %)	14 (3.0 %)	0.756
Wound treatment	68 (2.5 %)	54 (2.3 %)	14 (3.0 %)	0.392
Skin graft or flap	18 (0.6 %)	16 (0.7 %)	2 (0.4 %)	0.520
Limb amputation	1 (0.04 %)	1 (0.04 %)	0 (0.00 %)	0.654
Negative pressure wound therapy	8 (0.3 %)	6 (0.3 %)	2 (0.4 %)	0.525
Blood transfusion (number of patients)	85 (3.1 %)	61 (2.6 %)	24 (5.2 %)	**0.004** ^**a**^
In-hospital death	2 (0.07 %)	2 (0.09 %)	0 (0.00 %)	0.999
B.				
	Coefficient	95 % CI	*P*-value	
Length of stay (days)	−6.9	−10.0 to -3.7	**< 0.001** ^**b**^	
Hospitalization costs (dollars)	5531	4647–6417	**< 0.001** ^**b**^	
Costs of operation (dollars)	6577	6274–6881	**< 0.001** ^**b**^	
	Odds ratio	95 % CI	*P*-value	
Additional procedure during hospitalization	0.85	0.43–1.68	0.634	
Blood transfusion (number of patients)	1.40	0.77–2.53	0.270	

^a^The values in bold indicate significant differences at the 0.005 (=0.05/10) significance level

^b^The values in bold indicate significant differences at the 0.05 significance level

Table 4 shows the incidence of perioperative adverse events limited to the patients who underwent TAA. The overall rate of perioperative adverse events during hospitalization was 7.1 % (33 of 465). The rate of perioperative adverse events was higher in those with a Charlson Comorbidity Index of 1 or greater, those in the low-volume hospital group, and those with anesthesia time of 200 min or greater. Multivariable logistic regression analysis demonstrated that perioperative adverse events after TAA were independently associated with hospital volume and anesthesia time, with significantly reduced odds in high-volume hospitals (odds ratio, 0.31; 95 % confidence interval, 0.10–0.96; Table 5) and significantly increased odds in the group with longer anesthesia time (odds ratio, 2.83; 95 % confidence interval, 1.10–7.28; Table 5).

**Table 4:** Incidence of perioperative adverse events after total ankle arthroplasty in each subgroup

	Total	Perioperative adverse events	*P*-value
All	465	33 (7.1 %)	
Age (year)			0.071
≤ 39	4	1 (25.0 %)	
40–49	6	1 (16.7 %)	
50–59	56	8 (14.3 %)	
60–69	152	12 (7.9 %)	
70–79	197	8 (4.1 %)	
≥ 80	50	3 (6.0 %)	
Sex			
Male	76	6 (7.9 %)	0.767
Female	389	27 (6.9 %)	
Body mass index			
< 30	333	22 (6.6 %)	0.857
≥ 30	26	3 (11.5 %)	
Missing	106	8 (7.5 %)	
Smoking			
Nonsmoker	322	19 (6.0 %)	0.128
Ex or current smoker	35	4 (11.4 %)	
Missing	75	10 (13.3 %)	
Charlson Comorbidity Index			
0	423	26 (6.1 %)	**0.001**
≥ 1	42	7 (16.7 %)	
Mean hospital volume (per year)			
≤ 4	269	29 (10.8 %)	**< 0.001**
≥ 5	196	4 (2.0 %)	
Underlying diagnosis			
Osteoarthritis	286	13 (4.5 %)	0.007
Rheumatoid arthritis	179	20 (11.2 %)	
Anesthesia time			
< 200 min	270	7 (2.6 %)	**< 0.001**
≥ 200 min	195	26 (13.3 %)	

The values in bold indicate significant differences at the 0.006 (=0.05/8) significance level

**Table 5:** Multivariable logistic regression for perioperative adverse events after total ankle arthroplasty

	Odds ratio	95 % CI	*P*-value
Age (years)	0.970	0.932–1.010	0.140
Charlson Comorbidity Index			
0	1.00		
≥ 1	2.16	0.83–5.64	0.116
Mean hospital volume (per year)			
≤ 4	1.00		
≥ 5	0.31	0.10–0.96	**0.042**
Underlying diagnosis			
Osteoarthritis	1.00		
Rheumatoid arthritis	1.43	0.63–3.24	0.393
Anesthesia time			
< 200 min	1.00		
≥ 200 min	2.83	1.10–7.28	**0.031**

The values in bold indicate significant differences at the 0.05 significance level

## Discussion

Evidence of improved clinical outcomes has resulted in an increase in the number of TAAs performed worldwide for end-stage ankle arthritis. The present study found that TAA remains a less common procedure in Japan, and that low hospital volume was associated with an increased risk of complications after TAA.

The proportion of TAAs performed did not show any apparent increase between 2007 and 2013 in Japan, and only around 100 TAAs were performed annually after 2011. Considering the fact that the DPC database covers more than 50 % of all inpatient admissions to acute-care hospitals in Japan, it can be estimated that 0.2 TAA per 100,000 inhabitants is performed annually in Japan. This figure is fairly low compared with 1 TAA per 100,000 inhabitants in Sweden, Norway, and the United Kingdom [4, 5, 19], approximately 2 per 100,000 inhabitants in Finland and Germany [13, 20], 0.6 to 2.5 per 100,000 inhabitants in Australia and New Zealand [6, 21], and 1.9 to 4.0 per 100,000 inhabitants in the United States [3, 22]. The ratio of TAA to ankle arthrodesis was also low in the present study (1:6), compared with 1:2–3 in the United States, France, and Germany [3, 18, 20, 23]. These differences likely reflect preferences of surgeons in Japan to perform ankle arthrodesis over TAA compared with surgeons in Western countries.

The present study showed that the underlying diagnosis was significantly different between TAA and ankle arthrodesis groups, indicating the preference of surgeons to perform TAA over ankle arthrodesis for patients with RA in Japan. The percentage of RA as an underlying diagnosis among patients undergoing TAA was 38.5 % in the present study, which was comparable to that in Scandinavian countries [4, 5, 13]. In contrast, studies from Australia, New Zealand, the United Kingdom, France, and the United States demonstrated that the percentage of RA was less than or around 10 % [6, 19, 21, 24]. A previous study from the United States demonstrated a decrease in the percentage of RA from 10.8 % in 1998–2000 to 4.9 % in 2009–2010 [2]. This trend could indicate that growing evidence of good clinical outcomes after TAA has been expanding the indication for TAA from low-activity patients such as those with RA to higher-level activity patients. The higher percentage of TAA utilization in RA patients in Japan may imply that Japanese surgeons have a perception that the risks outweigh the benefits in high-activity patients and the benefits outweigh the risks in those with RA.

Both the total hospitalization costs and operative costs were significantly higher in the TAA group. This can be explained by the high implant costs associated with arthroplasty. The blood transfusion rate was significantly lower in the ankle arthrodesis group. This result is contradictory to a report that compared the perioperative complications and hospitalization outcomes after ankle arthrodesis and TAA using the data of the National Inpatient Sample in the United States, which has been the only study comparing the blood transfusion rate between the two groups [23]. We could not perform a multivariate analysis to adjust for differences in demographics and comorbidities according to blood transfusion because of the small number of events. Further accumulation of data will be needed to make a conclusion about this topic.

There are a limited number of studies comparing the perioperative complications and hospitalization outcomes between ankle arthrodesis and TAA. A previous study in the United States reported that TAA was independently associated with a decreased risk of overall complication during hospitalization [23]. Another study in the United States reported that patients treated with TAA had a significantly increased rate of major revision at 90 days postoperatively [25]. A multicenter, prospective, cohort study by the Canadian Orthopaedic Foot and Ankle Society comparing ankle arthrodesis and TAAs performed between 2001 and 2007 reported that the major complication rate was 7 % for ankle arthrodesis and 19 % for TAA [26]. Because of the difference in observational periods, definition of complications, and backgrounds of the patients, we could not directly compare the present study with previous ones. In particular, the duration of hospitalization in Japan is the longest among the Organisation for Economic Co-operation and Development countries [27], which might be attributed to factors including differences in healthcare systems and cultural norms. The duration of hospitalization of around 40 days after TAA in Japan (including the rehabilitation period) is far longer than that, for example, in the US, which is reportedly around 3 days [3, 22]. Owing to the longer duration of hospitalization in Japan, we consider that most of the perioperative complications were included in our analysis. We believe the present study is valuable in revealing that most of the additional procedures during hospitalization were related to wound complications and there was no significant difference in the rate of additional procedures required for these between TAA and ankle arthrodesis groups.

Wound issues are one of the common complications in both TAA and ankle arthrodesis, and can be a major problem, leading to implant infection and limb amputation especially after TAA [28]. A multicenter, prospective, nonrandomized, 2-phase comparison of ankle arthrodesis and Scandinavian Total Ankle Replacement (STAR) showed that major complications and the need for secondary surgical intervention were more common in the TAA group; however, the rate of major complications in the TAA group decreased in the second phase of the trial compared with the first phase [12], and this was attributed to increased surgeon experience and some modifications to the instruments and technique. As that study demonstrated, the rate of perioperative complications after TAAs procedure is also associated with the popularization of TAA. We believe that the present study provides important epidemiological information for future investigations.

Many factors have been considered risks for delayed wound healing in total joint arthroplasty, including previous incisions, lymphedema, poor vascular perfusion, obesity, diabetes mellitus, inflammatory arthropathy, renal or liver disease, immune compromise, corticosteroid therapy, smoking, poor nutrition, and a long operative time [29–31]; some of these have also been identified as risks in TAA. Raikin et al. reported that underlying inflammatory arthritis was the only significant risk factor for major wound complications using multivariate analysis [32]; however, RA was not identified as an independent risk factor in the present study. Because background and indication for surgeries in RA patients might differ between countries, direct comparison is difficult. Obesity and smoking are well-recognized risk factors for delayed wound healing after TAA [33–36], although these factors were not identified as risks in the present study. We think that this inconsistency might be attributed to the small number of subjects in the present study. Kessler et al. reported that patients with wound healing problems were at risk for infection of TAA, and patients with periprosthetic ankle joint infection had longer operative times than matched controls [28]. The present study demonstrated that longer anesthesia time was independently associated with perioperative complications. We could not obtain the operative time from the DPC database, and so we substituted anesthesia time as a reflection of the operative time.

Some studies suggested that low-volume centers were associated with implant failures or poor survival rate [18, 37]. In France, the National Commission for the Evaluation of Medical Devices and Healthcare Technologies proposed to limit TAA to centers that performed at least 10 TAAs per year for the past 3 years [18]. The present study demonstrated that a higher hospital volume was independently associated with a lower rate of perioperative complications after TAA. A study from France, which has been the only study analyzing the effect of hospital volume on outcome after TAA, reported that infectious and cutaneous complications were more likely to occur in high-volume centers (≥ 5 cases/year) compared with the low-volume centers (≤ 4 cases/year) (16 % vs 5 %, *P* = 0.015) [18]. They included only 97 cases performed in 3 high-volume centers and 86 cases in 6 low-volume centers. We believe our study is advantageous because of its large sample from a national database. Given the technical demands and experience required to perform TAA successfully, the current situation in Japan that most hospitals perform < 5 TAAs annually raises concerns about provider competence. Moreover, the dispersion of a relatively low number of TAA cases over many hospitals may make the investigation of clinical outcomes difficult, and may be one of the reasons for the slow growth of TAA in Japan. Another possible reason for the slow growth of TAA is the limited availability of marketed TAA designs. Only 2 implants are available in Japan: the TNK ankle (Kyocera Medical, Osaka, Japan) and the FINE total ankle system (Teijin Nakashima Medical, Okayama, Japan). The numbers of different implant designs available in other countries are 10 in Australia, 7 in the UK, 6 in the United States, New Zealand, and Sweden, and 5 in Finland and Norway [38]. In addition, we have demonstrated that the lower the number of TAA cases per hospital, the more susceptible the TAA procedure is to complications. This may make it more difficult for surgeons to opt for TAA. To make it possible for the public to access this therapeutic option, further efforts will be required by Japanese foot and ankle surgeons to investigate the long-term clinical outcomes of Japan-originated TAA implants. A joint registry system or centralization of TAA cases at a restricted number of facilities should be considered as one means for solving the problem. Moreover, creating opportunities to obtain additional specialized training for surgeons aiming to perform TAA would be desirable to compensate for this lack of experience.

Several limitations of our study must be acknowledged. First, the use of an administrative claims database could have led to underestimation or overestimation of backgrounds or perioperative complications because of incomplete reporting or misclassification. Although we were unable to verify the data for each patient, we presume that there was no difference in the proportion of miscoding between the TAA and ankle arthrodesis groups, and that the level of miscoding is low if any, as the DPC data are coded by physicians and subject to an audit. Second, the DPC database is less likely to reflect the situation in small hospitals because hospitals participating in the database are relatively large. Third, the DPC data was not obtained for the entire year between 2007 and 2010. However, we believe that there may not be any selection bias, as there is presumably no seasonal variation in the surgical procedures evaluated in this study. Fourth, although surgeon volume might also affect the perioperative complications, this information was not able to be obtained and included in the analyses. However, most of the perioperative complications were assumed to be included in our analysis as the mean hospital stay was around 40 days in both groups. Despite these limitations, we believe that the present study is epidemiologically important because it demonstrates trends in the use of ankle arthrodesis/TAA and compared backgrounds and perioperative complications comprehensively between these two procedures for the first time in the Japanese population.

## Conclusions

In summary, the proportion of TAA procedures did not markedly increase from 2007 to 2013 in Japan, in contrast to an increase reported in other developed countries. Lower hospital volume and anesthesia time were associated with higher rates of perioperative adverse events after TAA.

## Abbreviations

DPC: Diagnosis procedure combination
ICD-10: International Classification of Disease, Tenth Revision
OA: Osteoarthritis
RA: Rheumatoid arthritis
TAA: Total ankle arthroplasty
